# An Analysis of Lidocaine Usage in the Treatment of Squamous Cell Carcinoma

**DOI:** 10.7759/cureus.35614

**Published:** 2023-02-28

**Authors:** Maya Maalouf, Akshay J Reddy, Pasha Mazboudi, Mildred Min, Rushil Rawal, Casey A Curow, Monica E Guirgus, Danny S Abdalnour, Telak Brahmbhatt, Rakesh Patel

**Affiliations:** 1 Medicine, California University of Science and Medicine, Colton, USA; 2 Dermatology, California Northstate University College of Medicine, Elk Grove, USA; 3 Health Sciences, California Northstate University, Rancho Cordova, USA; 4 Internal Medicine, East Tennessee State University, Quillen College of Medicine, Johnson City, USA

**Keywords:** mohs surgery, xylocaine, cancer treatment, squamous cell carcinoma, lidocaine

## Abstract

Squamous cell carcinoma (SCC) is a form of skin cancer that can be treated using a procedure known as Mohs surgery. Mohs surgery is a safe and effective procedure for eliminating SCC. This surgery requires the usage of an analgesic known as lidocaine. Additional anesthetics were also reported to be necessary for this procedure to be conducted in a manner that significantly minimizes patient harm. According to the review, it was found that SCC was treated with lidocaine as a topical analgesic outside of Mohs surgery. This review analyzes the usage of lidocaine in the treatment of SCC. It was also discovered that lidocaine, as an agent, has the potential to slow the progression of SCC, but more research is needed to see if this is truly the case. On average, it was reported that the concentration of lidocaine used in the in vivo studies was significantly higher than that in the in vitro investigations. Further exploration may be needed to verify the conclusions that were based on the analysis of the papers within the review.

## Introduction and background

Squamous cell carcinoma (SCC), a non-melanoma skin cancer, is a keratinocyte carcinoma and is one of the most prevalent malignancies with a rising incidence (SCC of the anus incidence rates increases nearly 3% each year) [1,2]. In fact, it is anticipated that approximately 700,000 new instances of cutaneous squamous cell carcinoma (CSCC) are detected annually in the United States [3]. The accumulated exposure of skin to UV light culminates in SCC, the second most prevalent form of skin cancer. Age, cumulative sun exposure, pale skin, continuous immunosuppression, and past skin cancer diagnoses are significant SCC risk factors [3-5]. This illness is characterized by precursor lesions known as actinic keratosis, tumor growth (typically greater than 3 cm in size at stage IV SCC and less than 3 cm at earlier stages), and the potential for metastasis inside the body. SCC is responsible for the majority of non-melanoma skin cancer-related metastatic illnesses; consequently, early detection and treatment of SCC are crucial for preventing neoplastic development [4,6]. The prognosis for the majority of patients with primary SCC is favorable, and treatment is typically easy. However, a sizable proportion of malignant neoplasms may return or metastasize. On average, SCC tumors are reported to have a diameter of approximately 1.5 cm [3]. Surgical excision is the primary treatment for CSCC, with Mohs micrographic surgery becoming a preferred excisional procedure for SCC of the neck and head as well as other areas with high-risk or SCCs with high-risk features [6]. Radiation therapy (shown to have a 90% five-year cure rate) is reserved for elderly patients with SCC, for those who cannot endure surgery, or when it has been impossible to acquire clear surgical margins [6,7]. On average, patients who receive radiation therapy for SCC have a survival rate of approximately 89% if treated in the early stages [3]. Very high tumors are typically treated with adjuvant radiation following surgical intervention. Mohs surgery necessitates the use of lidocaine as an anesthetic. In vitro studies of SCC cells have also utilized lidocaine [5-6]. The objective of this review is to examine how lidocaine contributes to SCC treatment and our current understanding of the disease.

## Review

Methods

**Figure 1:**
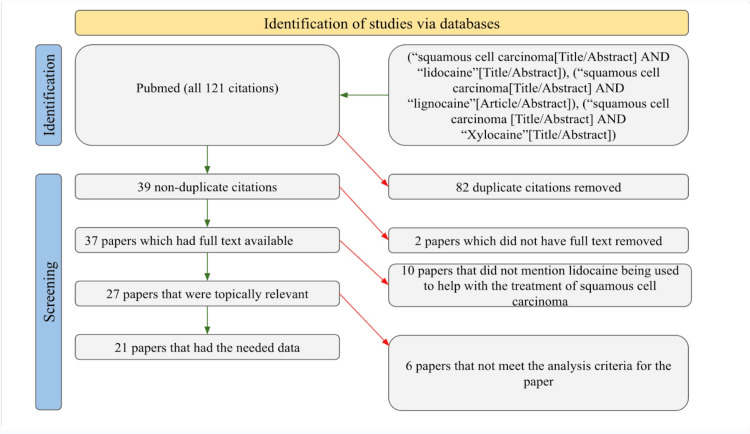
PRISMA diagram representing the study selection process PRISMA: Preferred Reporting Items for Systematic Reviews and Meta-Analyses.

We conducted a search on PubMed to find studies about the use of lidocaine in the treatment of SCC. Exact searches were done with the keywords “squamous cell carcinoma and lidocaine,” “squamous cell carcinoma and lignocaine,” and “squamous cell carcinoma and xylocaine.” We placed no restrictions in terms of time frames in the search. The search elicited a total of 121 studies, of which 39 were not duplicates, 37 had full text available, 27 were topically relevant studies, and only 21 had the relevant information needed and fulfilled the analysis criteria of our review. The data collected from these 21 studies included the dosage of the lidocaine used, the area of anesthetic application, additional medications used, and the route of administration. Studies that did not provide sufficient information about at least two of these categories were excluded. This was done to avoid personal bias. Figure 1 provides a clear illustration of the filtering procedure used by the authors of this review.

Mohs surgery

While there are several different treatment options for SCC based on the progression of the disease, Mohs surgery, also known as Mohs micrographic surgery, is the best option in many cases due to its advantages in removing the cancerous region. Mohs surgery holds the highest cure rate of any SCC treatment [4-7]. Although there are other treatment options such as wide local excisions, electrodessication, and curettage which are viable and effective treatment options, the accepted maximum cure rate is approximately 95%, whereas Mohs is able to achieve a higher cure rate of 99% [4-7]. The reason for the discrepancy lies in the way the procedure is conducted. The physician ensures clear margins with microscopic examinations at the time of treatment versus sending the specimen to a dermatopathologist for inspection as with wide local excisions [8-10]. Thus, the entire cancerous region is removed at once, with a very minimal risk of disease progression and no additional risk of complications from a second procedure if the margins are not clear. Mohs surgery not only has the highest cure rate, but it also has the best patient outcome for SCC removal. The studies that were analyzed, as shown in Table 1, have data that also seems to support this hypothesis [8-28]. With the stage-wise protocol of tissue removal of cancerous tissue until clear margins are seen, Mohs surgery minimizes the amount of healthy tissue being removed. This precision is especially important for sensitive areas like the face and nose, where it is not only important for positive patient satisfaction but also for optimal surgical site closure to minimize scarring [6]. Further, since the procedure is done under local anesthesia, there is a reduced risk of complications that may arise from general anesthesia, making it a safer treatment option.

According to the data presented in Table 1, aside from lidocaine, ketamine was the most frequently administered drug during Mohs surgery for SCC [8-28]. Low-dose perioperative ketamine has been found to alleviate postoperative pain and reduce the use of opioids when lidocaine failed to provide relief [12-15]. Increasingly, ketamine and lidocaine have been used in medicine together. Currently, ketamine is prescribed for anesthesia, pain, and intensive care. Adults and children are sedated with ketamine as the literature strongly supports the safety and efficacy of ketamine for dissociative sedation in adults and children in an effort to comfort and reduce anxiety and pain during painful or distressing procedures [9-12]. Ketamine administered to patients in the intensive care unit provides a combination of sedation and analgesia, favorable hemodynamic effects, and the ability to treat persistent bronchospasm. Small subanesthetic doses of ketamine have been administered topically or intravenously as an analgesic for the treatment of chronic pain. While ketamine has shown beneficial effects when it comes to analgesia and pain, there are certain side effects associated with its use [13-17]. Patients who received ketamine during surgery were more likely to experience hallucinations and nightmares in the recovery room and for several days following surgery. Due to its unique pharmacological benefits and newly discovered clinical properties, ketamine has multiple clinical applications. In addition to anesthesia, ketamine is now used for pain, palliative care, intensive care, and procedural sedation. It is increasingly administered in low doses and in conjunction with other drugs.

**Table 1:** The usage of lidocaine in the treatment of squamous cell carcinoma

Author (year)	Reported Dosage or Concentration of Lidocaine	Area of Application	Additional Pharmaceutical Medications	Route of Administration
Clark and Kalan (1995) [9]	100 mg	Intravenous	Ketamine	In vivo
Ferreira et al. (2018) [10]	1-5%	Applied to cells (incubation)	Doxorubicin	In vitro
Firoz et al. (2009) [11]	0.50%	Injection (type unspecified) into digits	Aspirin, coumadin, plavix, and vitamin E	In vivo
Hakim et al. (2018) [12]	1, 1.5, 2 mg/kg	Intravenous	Ketamine, lidocaine, mexiletine, methadone, and morphine	In vivo
Heller et al. (1998) [13]	1%	Injection (type unspecified) around the treatment site	Bleomycin	In vivo
Ho et al. (2004) [14]	2%	Topical (on tongue/pharynx) followed by gentle suctioning	Atropine	In vivo
Johnstone et al. (1995) [15]	2.50%	Case 1: Intravenous; Case 2: Intravenous, then topical; Case 3: Intravenous, then topical; Case 4: Intravenous, then topical	Nitrous oxide isofluoride fentanyl	In vivo
Kintzel et al. (2018) [16]	1 mg/minute (60 mg/hour), 0.8 mg/minute (48 mg/hour), 0.6 mg/minute (36 mg/hour), 0.6 mg/minute (36 mg/hour), 0.4 mg/minute	Intravenous	Gabapentin, methadone, ketamine acetaminophen, and pro re nata (PRN) hydromorphone	In vivo
Kobayashi et al. (2012) [17]	400 μM, 676.6 μM, 735.5 μM, 811.6 μM, and 4000 μM	Applied to cells	Dibucaine tetracaine, bupivacaine, lidocaine, and procaine	In vitro
Krishnan and Mitragotri (2020) [18]	N/A	N/A	5-FU, imiquimod, and ingenol mebutate	N/A
Lee et al. (2013) [19]	N/A	Topical	Papaverine, streptokinase, and urokinase	In vivo
Liu et al. (2022) [20]	0, 1,5, and 10 mM	Applied to cells	Cisplatin	In vitro
Mücke et al. (2015) [21]	20 mg/g lidocaine hydrochloride	Oral (salve)	Diclofenac and omeprazole	In vivo
Sercarz et al. (1995) [22]	1%	Injection (nerve)	N/A	In vivo
Strickland et al. (1993) [23]	100 mg	Intravenous	Fentanyl citrate, nitrous oxide, isoflurane fentanyl, and atropine	In vivo
Tartaglione et al. (2008) [24]	10%	Topical (spray)	Nanocolloidal	In vivo
Thakur et al. (2012) [25]	Three subsequent sprays of 7%, followed by an intratracheal injection of 5%	Topical (spray) followed by intratracheal injection	N/A	In vivo
Turnbull et al. (2011) [26]	Patch 5%, jelly 2%	Topical (jelly)	Bisphosphonate, ropivacaine, fentanyl, ketamine, morphine, methadone, oxycodone, haloperidol, and mepivacaine	In vivo
Wang et al. (2016) [27]	5%	Applied to the lip (assuming topical)	Cefuroxime axetil, prednisone acetate, and ketotifen fumarate	In vivo
Wiese et al. (1993) [29]	0.5 ml 0.25 %, 7 μg/mL	Applied to cells: Injection (single dose) or medium with lidocaine	N/A	In vitro
Yasuta et al. (2014) [31]	1%	Intradermal	Iomeprol (as a contrast agent)	In vivo

Lidocaine usage

Topical administration of lidocaine at a mean initial dosage of 1 mg/kg/h (range: 0.5-2.7 mg/kg/h) was found to be the most common route of application in the in vivo studies investigating SCC [8-29]. In these studies, lidocaine was administered topically to the tongue and pharynx, oral cavity, lip arm, or scalp [13,23-26]. Based on the areas being treated, it is reasonable to find that lidocaine was applied topically for the following reasons. One is that topical administration locally numbs the area of application. Presumably, this remains a safer route with fewer side effects (lightheadedness, dizziness, blurred vision, low blood pressure, etc.) than other types of anesthesia (e.g., intravenous or intradermal) due to its localized mechanism of action. Topical administration of lidocaine also results in a shorter duration of nerve blocking, which provides a shorter recovery time for patients [14-22,30]. As a result, topical lidocaine would be favored for shorter procedures to reduce the likelihood of severe postoperative symptoms and complications. It is important to highlight, however, that the administration route used is dependent upon the area and size of the application site. Topical lidocaine will not be preferred in all situations. Lastly, regarding the in vitro studies found, lidocaine was administered topically in all studies [8-28]. Cells were incubated with lidocaine in various concentrations to stimulate and test the effects of this anesthetic [9,16]. Logically, topical lidocaine remains the only possible route of administration for in vitro studies. Several papers analyzed within this study applied lidocaine directly to squamous cells rather than conducting an in vivo study. According to the data presented in Table 2, the variance of lidocaine concentration among in vivo studies was found to be higher than that in the in vitro studies.

**Table 2:** Variance and mean concentration of lidocaine

Group	Variance	Mean Concentration
In vivo	8.704	3.10%
In vitro	7.44	0.01%

Through analysis, it can be observed that all these papers were aiming to observe the cytotoxic effects caused by the combined effects of cisplatin and lidocaine on squamous cells [9,16]. Approximately 90% of SCC cases are seen within the oral cavity [9,31]. Therefore, this paper aimed to test the effects of lidocaine through an in vitro study that applied lidocaine to cell lines from human tongue SCCs. Local anesthetics are commonly applied to tumors during head and neck surgeries; however, their effects on the oral cavity are unknown. One study compared seven local anesthetics, one of them being lidocaine, and looked at their effects on oral tumor cells relative to normal cells [16]. This study was conducted with the aim to visualize the effects of various anesthetics on the proliferation of SCC cells. One of the studies in the review also analyzed the cytotoxic effects of lidocaine on the growth of cells within head and neck SCCs. This study specifically looked at the effects of lidocaine on spindle cells and round cells found within SCC of the head and neck [27]. Future studies may be conducted to further understand the mechanisms by which lidocaine exerts its cytotoxic effects. According to the data presented in Tables 2, 3, the mean concentration of lidocaine used in the in vivo studies was significantly higher than the dosage used in in vitro studies. Concentration was compared by the same standard by converting molar to percent. This may be due to the fact that eliciting a chemical response from a human body requires a higher concentration than plated cells due to their significantly larger mass.

**Table 3:** Statistical significance between group means

Group Comparison	T-value	P-value
In vivo versus in vitro anesthesia concentration	3.64	0.015

Future applications and current limitations

The results from the culmination of 21 studies suggest lidocaine use for patients with SCC undergoing Mohs surgery. There are no trends in the dosage of lidocaine as there is such a wide range. In many studies, lidocaine was combined with a secondary medication. Medications used in multiple studies include fentanyl, ketamine, nitrous oxide, cisplatin, capsaicin, and atropine. The results from this review can help physicians understand that there is a myriad of concomitantly administered drugs with lidocaine. The lack of trends gives an insight into the variability in the approach to lidocaine usage, as instead of it appearing as a standardized approach to care, it is illustrated as a patient-to-patient decision made by physicians. This review was able to effectively examine the effects of lidocaine application and Mohs surgeries. Through analysis of 21 studies, we were able to analyze the effects of different dosages and applications of lidocaine on SCC patients and cell lines. One of the limitations of this review was that several studies indicated the use of additional pharmaceutical medications, while others did not. This might introduce confounding variables between different papers. Similarly, the use of different additional medications might have resulted in different outcomes between the studies. Another limitation is that the majority of studies reported using topical or injected lidocaine, while only a few mentioned using the lidocaine orally or via application to cells. This discrepancy might account for any variable results across the many procedures. Therefore, future studies could consider this aspect and examine the different methods of lidocaine application equivalently. One way to improve this might be to conduct a review of studies that used topical, oral, cell application, intravenous, and intradermal lidocaine application equally to remove any confounding variables from the results. With these limitations in mind, lidocaine and Mohs surgery could be evaluated further. Despite the extensive study that has been undertaken on the administration and use of lidocaine on patients with SCC, new research is required to find more optimal standards that may be employed for these operations in order to avoid any side effects.

Currently, we are aware that the normal concentration of lidocaine during Mohs surgery is 1%; however, we do not have a process or procedure to follow if this concentration needs to be modified to fit the needs of a particular patient. Additional studies utilizing combination agents may be necessary to address these issues. In order to investigate the effects of various substances on the analgesic effects of lidocaine, it may be necessary to use chemicals such as ethylenediaminetetraacetic acid (EDTA) during this research. It is crucial that individuals with SCC receive an early diagnosis to minimize adverse health problems and diminish returns on treatment. This will help alleviate the discomfort that patients may experience as a result of Mohs surgery. AI software that examines dermatomes may be developed in the future to expand the number of SCC patients who are diagnosed. It is vital to conduct further studies on lidocaine's side effects to ensure the safety of SCC patients undergoing Mohs surgery.

## Conclusions

This review was conducted to evaluate the usage of lidocaine as an anesthetic in the treatment and understanding of SCC. Based on the data collected from the literature, a statistical analysis was conducted, and the results suggested that the concentration of lidocaine used to create an analgesic effect on patients undergoing Mohs surgery was higher than the concentration of lidocaine that was necessary to slow the growth of SCC cells. Further investigations should be conducted to validate this finding. Additionally, more research is necessary to understand the potential side effects of using lidocaine in the treatment of SCC.
